# Animal Temperature under Narcotics

**Published:** 1894-02-03

**Authors:** Benjamin Ward Richardson


### Animal Temperature Under Narcotics.

By Sir Benjamin Ward Richardson, M.D., F.R.S.

In my last article the fact that the animal temperature falls and may fall to a fatal degree under the influence of chloral hydrate was specially dwelt upon, and was made the subject of a particular lesson bearing on the treatment that ought to be adopted after that narcotic has been taken in a poisonous dose. I pass from this fact to another of a broader stamp; I pass to note, from direct observation, that fall of temperature attends the action of various other narcotics with which we are very familiar.

The phenomenon of decline of animal heat follows, as far as I am aware up to the present time, the full administration of all narcotic substances with one grand exception, namely, opium and its derivatives. This marks out a most important rule, namely: When a narcotic is demanded, and the animal temperature is normal, or is below the normal, prescribe an opiate; but when there is pyrexia avoid opium, or combine it with some other agent or agents that have a counteracting effect, in so far as elevation of temperature is concerned. It is from the empirical observance of this rule that certain combinations of opium with other drugs have proved so beneficial in febrile diseases, Dover's powder being, of all time-honoured remedies, the best example that could be presented in this respect. As an addendum, another rule may safely be offered, namely: Whenever in the course of disease a narcotic is demanded, the animal temperature being above the normal, always prescribe a narcotic that has a tendency to reduce the febrile condition.

The first of these rules is most easily under our command, for where it is applicable opium is the prince of narcotics. More than that, it is better than any other drug when the animal temperature is low. It gives no headache then, produces no troublesome constipation, causes neither nausea nor vomiting, acts like a food, and, at the same time, relieves pain and tends to produce sleep. But when we come to the study of what may be called febrifuge narcotics, the scene changes; there are now great differences before us, and at present considerable perplexities. We have before us a new, I may say an almost unexplored field; a field in which, as far as I am aware, there has been no labourer besides myself in the clinical as well as physiological department; and my own labours are, so small they do but turn the first sod.

I commenced my inquiries by a study of the chlorides of the organic group, and amongst these chloroform came naturally as the first for investigation. I noticed as far back as the year 1852 that during the administration of chloroform there is a declining temperature in the period of inhalation, and I tested this observation on a considerable number of the inferior warm-blooded animals. The test proved most satisfactorily corroborative. I began next to employ the same remedy in the treatment of acute febrile disease, and with so much success that I thought at one time I had succeeded in causing some of these diseases to abort, or become checked at an early stage. I was, perhaps, premature in this hope, but the results were sufficient to lead me to a more determinate experimental inquiry, which brought out many new and uniform truths. In order to arrive at correct data, a period of three hours was devoted to each observation, so that no accidental interference should lessen the value of the observation. The air of the room was sustained at a steady and uniform warmth, 64 deg. Fahr. Three observers were all the time at work, and when there was any doubt there was careful repetition of the observation. The temperature of the animal was taken before the administration of the narcotic, as well as at various stages during the time of the narcotism; and, the animals subjected to the narcotism, a warm-blooded animal in every case, were such as showed as wide a difference as possible in natural range of animal warmth, from the guinea-pig with a normal of 101 deg. Fahr., to the pigeon with a normal of 109 deg. Fahr. Suffice it to add that the animals were put to sleep with the same care and quiet as if they had been human beings about to undergo a surgical operation painlessly; that they were all healthy, and that none were injured, or subjected to any touch of pain.

The effect of the administration up to complete narcosis was always to produce a fall of the animal temperature. During the convulsive stage which, in the human subject, marks what is called the second stage, there was a slight rise amounting to nearly a degree, as if the friction of the muscles upon each other caused an elevation; this over the effect was invariably decline of animal warmth, and to such an extent that in one bird it was no less than a decline of six degrees, attended with the signs of impending death, namely, feeble breathing, intermitting pulse and complete muscular prostration. Recovery of temperature commenced, I observed, so soon as the symptoms of narcotism began to pass away, but when the reduction had been brought to the lowest a considerable interval of time elapsed before the full normal temperature was re-established. In another series of inquiries I endeavoured to learn what would be the effect on the animal temperature of the administration of chloroform if narcotism were induced in an atmosphere where the air was itself raised, as in a Turkish bath. I found that in the pigeon the temperature of the body rose to 112 deg. Fahr. in a temperature of 120 deg. when it remained stationary, In this elevated temperature, so soon as the first and second stages of excitement from inhalation was past, and narcosis was established, the temperature of the bird began to fall until it came down to 107 deg., and then suddenly to 105 deg., when insensibility was profound. On withdrawing the vapour and permitting recovery the animal warmth rose again much more quickly than when the reduction had been produced in a cooler air.

Conversely I learned that by producing the narcotism in combination with external cold the animal warmth in the bird could be brought down to 97 deg. Fahr.—thirteen degrees—a reduction which is probably the lowest compatible with life, and from which recovery to the normal is long delayed. These phenomena regarding the systematic fall of animal temperature are basic in their bearings on the use of what are called antipyretics.

